# The Vertex Version of Weighted Wiener Number for Bicyclic Molecular Structures

**DOI:** 10.1155/2015/418106

**Published:** 2015-11-10

**Authors:** Wei Gao, Weifan Wang

**Affiliations:** ^1^School of Information Science and Technology, Yunnan Normal University, Kunming 650500, China; ^2^Department of Mathematics, Zhejiang Normal University, Jinhua 321004, China

## Abstract

Graphs are used to model chemical compounds and drugs. In the graphs, each vertex represents an atom of molecule and edges between the corresponding vertices are used to represent covalent bounds between atoms. We call such a graph, which is derived from a chemical compound, a molecular graph. Evidence shows that the vertex-weighted Wiener number, which is defined over this molecular graph, is strongly correlated to both the melting point and boiling point of the compounds. In this paper, we report the extremal vertex-weighted Wiener number of bicyclic molecular graph in terms of molecular structural analysis and graph transformations. The promising prospects of the application for the chemical and pharmacy engineering are illustrated by theoretical results achieved in this paper.

## 1. Introduction

The past 35 years have witnessed the conduction of investigations of degree or distance based topological indices. Topological indices are numerical parameters of molecular graph, which play a significant role in disciplines such as physics, chemistry, medicine, and pharmacology science. The vertex-weighted Wiener number, as the extending version of Wiener index, reflects both the melting point and boiling point of chemical compounds (see Wiener [1] and Katritzky et al. [2] for more details).

Specifically, let *G* be a molecular graph; then a topological index can be regarded as a score function *f* : *G* → *ℝ* ^+^, with this property that *f*(*G*_1_) = *f*(*G*_2_) if *G*_1_ and *G*_2_ are isomorphic. Topological indices', which function as numerical descriptors of the molecular structure obtained from the corresponding molecular graph, several applications have been found in theoretical chemistry, especially in QSPR/QSAR study. For instance, scholars introduce Wiener index, Zagreb index, harmonic index, and sum connectivity index to reflect certain structural features of organic molecules. There were several papers contributing to determine these distance-based indices of special molecular graph (see Yan et al. [3], Gao and Shi [4], Gao and Wang [5], Xi and Gao [6], and Gao et al. [7] for more detail). The notation and terminology that were used but were undefined in this paper can be found in [8].

The molecular graphs that have been considered in this paper are of simplicity and of relevance. The vertex and edge sets of *G* are denoted by *V*(*G*) and *E*(*G*), respectively. One terminology issue we should clarify here is that the term “molecular graph” represents a chemical structure in many articles, whereas, in our paper, we consider the extremal vertex-weighted Wiener number of bicyclic molecular graphs, and the bicyclic molecular graph which reaches the minimum vertex-weighted Wiener number may not represent a real molecular structure in chemical sciences. From this point of view, we relax the restricted, and the term “molecular graph” represents all graphs in our paper. The Wiener index of molecular graph *G* was defined as

(1) $$\begin{array}{c} W\left(\;G\;\right)=\underset{\left\{u,v\right\}\subseteq V\left(G\right)}{\sum }d\left(\;u,v\;\right), \end{array}$$

where *d*(*u*, *v*) is the distance between *u* and *v* in *G*.

Several Wiener related indices for molecular structures were introduced. For example, as the extension of Wiener index, the modified Wiener index is denoted by

(2) $$\begin{array}{c} W_{\lambda }\left(\;G\;\right)=\underset{\left\{u,v\right\}\subseteq V\left(G\right)}{\sum }d^{\lambda }\left(\;u,v\;\right), \end{array}$$

where *λ* is a real number. Moreover, the hyper-Wiener index was defined as

(3) $$\begin{array}{c} WW\left(\;G\;\right)=\underset{\left\{u,v\right\}\subseteq V\left(G\right)}{\sum }\frac{d^{2}\left(u,v\right)+d\left(u,v\right)}{2}. \end{array}$$

The (singly) vertex-weighted Wiener number of molecular graph *G* was introduced by Došlić [9] as

(4) $$\begin{array}{c} W_{v}\left(\;G\;\right)=\underset{\left\{u,v\right\}\subseteq V\left(G\right)}{\sum }\frac{d\left(u\right)+d\left(v\right)}{2}d\left(\;u,v\;\right). \end{array}$$

This index has been shown to be strongly correlated to both the melting point and boiling point of the compounds. One important thing we should emphasize here is that the (general) weighted Wiener index was introduced by Klavžar and Gutman [10] which is stated as *W*(*G*, *w*) = (1/2)∑_*u*,*v*∈*V*(*G*)_ *w*(*u*)*w*(*v*)*d*_*G*_(*u*, *v*), where *w* : *V*(*G*) → *ℕ* ^+^ is a weighting function. Hence, formula (4) introduced by Došlić [9] is a special case of it where the weighting function is appropriately selected.

Several papers have contributed to the Wiener index and related numbers. Klavžar and Rho [11] studied the Wiener index of generalized Fibonacci cubes and Lucas cubes. Gutman et al. [12] determined a congruence relation for Wiener index and Szeged index. Fuchs and Lee [13] derived asymptotic expansions of moments of the Wiener index and showed that a central limit law for the Wiener index is established. Das et al. [14] compared the Wiener index, Zagreb indices, and eccentric connectivity index for trees. Klavžar and Nadjafi-Arani [15] presented several bounds for Wiener index and the Szeged index for connected molecular graphs. Mukwembi and Vetrík [16] obtained the sharp upper bounds for the Wiener index of trees with diameter at most 6. Knor et al. [17] yielded the relationship between the edge-Wiener index and the Gutman index for molecular graphs. Alizadeh et al. [18] presented explicit formulas for the edge-Wiener index for suspensions, bottlenecks, and thorny graphs. Dankelmann et al. [19] determined upper bounds of Wiener index in terms of the eccentric connectivity index. A. Ilić and M. Ilić [20] described a linear time algorithm for computing generalized Wiener polarity index and generalized terminal Wiener index for trees and partial cubes and characterized extremal trees maximizing these indices among all trees of given vertex number.

Although several advances have been made in Wiener index, Zagreb index, PI index, hyper-Wiener index, and sum connectivity index of molecular graphs, the study of vertex-weighted Wiener number for special chemical structures has been largely limited. In addition, bicyclic related structures are widely used in medical science and pharmaceutical field for their function as widespread and critical chemical structures. For example, bicyclic molecular graph is one of the basic chemical structures that exists widely in benzene and alkali molecular structures. Because of these, tremendous academic and industrial interests have been attracted to research the vertex-weighted Wiener number of this molecular structure from a mathematical point of view.

In this paper, we study the minimum vertex-weighted Wiener number of bicyclic molecular graphs.

## 2. Notations and Useful Lemmas

In what follows, we set |*V*(*G*)| = *n* and |*E*(*G*)| = *m* as the number of vertex and edge in molecular graph *G*, respectively. A leaf is a vertex of degree one, a stem is a vertex adjacent to at least one leaf, and pendant edges are edges incident to a leaf and stem which is denoted simply by *K*_2_. We set *P*_*n*_, *C*_*n*_, and *K*_1,*n*−1_ as the path, cycle, and the star with order (vertex number) *n*, respectively.

Let *c*(*G*) = *m* − *n* + 1 be a cyclomatic number of a connected molecular graph *G*. A molecular graph *G* with *c*(*G*) = *k* is called a *k* cyclic molecular graph. We specifically call *G* as a bicyclic molecular graph if *c*(*G*) = 2. Let *ℬ*(*n*) be the set of all bicyclic molecular graphs with *n* vertices, and there are two basic cycles *C*_*p*_ and *C*_*q*_ in each graph *G* ∈ *ℬ*(*n*). All the molecular graphs in *ℬ*(*n*) can be divided into three classes according to the relationship between the two basic cycles:

(i) Θ(*p*, *q*)⊆*ℬ*(*n*) is the set of bicyclic molecular graphs such that two cycles *C*_*p*_ and *C*_*q*_ have only one common vertex.
(ii) Γ(*p*, *q*)⊆*ℬ*(*n*) is the set of bicyclic molecular graphs such that two cycles *C*_*p*_ and *C*_*q*_ have no common vertex.
(iii) *Ω*(*p*, *q*, *l*)⊆*ℬ*(*n*) is the set of bicyclic molecular graphs such that *C*_*p*_ and *C*_*q*_ have a common path of length *l* ≥ 1.

**Figure 1:**
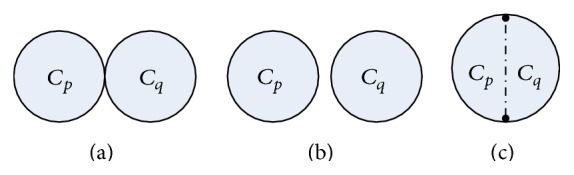
The structure of Θ(*p*, *q*), Γ(*p*, *q*), and *Ω*(*p*, *q*, *l*).

The structures of three classes of bicyclic graph Θ(*p*, *q*), Γ(*p*, *q*), and *Ω*(*p*, *q*, *l*) are manifested in Figures 1(a), 1(b), and 1(c), respectively. Furthermore, we have *Ω*(*p*, *q*, *l*) = *Ω*(*p*, *p* + *q* − 2*l*, *p* − *l*) = *Ω*(*p* + *q* − 2*l*, *q*, *q* − *l*).

Let *E*′⊆*E*(*G*) (or *W*⊆*V*(*G*)); we denote the subgraph of *G* obtained by deleting the edges (vertices) or *E*′ (or *W*) as *G* − *E*′ (or *G* − *W*). For any two molecular graphs *G*_1_ and *G*_2_, we denote this molecular graph as *G*_1_ *vG*_2_ if there exists a common vertex *v* between them. We denote it as *G*_1_ *uvG*_2_ if there exists a bridge *uv* between *G*_1_ and *G*_2_ with *u* ∈ *V*(*G*_1_) and *v* ∈ *V*(*G*_2_). If there are copies of molecular graphs *G*_1_, *G*_2_,…, *G*_*l*_ (*l* ≥ 2) with all molecular graphs sharing one common vertex *v*, this molecular graph is denoted by *G*_1_ *vG*_2_ *v* ⋯ *vG*_*l*_.

We will introduce some molecular graph transformations, which will decrease the vertex-weighted Wiener number of molecular graphs.

**Figure 2:**
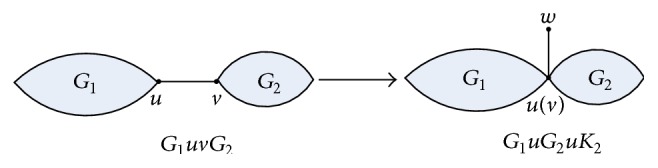
Transformation A.

*Transformation A*. Let *G*_1_ and *G*_2_ be 2-edge-connected molecular graphs with |*V*(*G*_1_)| ≥ 2 and |*V*(*G*_2_)| ≥ 2, and *uv* be an edge of *G*_1_ *uvG*_2_. *G*_1_ *uG*_2_ *uK*_2_ is the molecular graph transformed by *G*_1_ *uvG*_2_ using Transformation A, described in Figure 2.

Now, as preparation knowledge, we present some useful lemmas.

**Lemma 1 .**

Let *G*_1_ *uG*_2_ *uK*_2_ be a molecular graph obtained from *G*_1_ *uvG*_2_ by Transformation A; then *W*_*v*_(*G*_1_ *uG*_2_ *uK*_2_) < *W*_*v*_(*G*_1_ *uvG*_2_).

**Proof**

For convenience, we set *G*_1_^*∗*^ = *G*_1_ *uvG*_2_, *G*_2_^*∗*^ = *G*_1_ *uG*_2_ *uK*_2_, *G*_1_′ = *G*_1_ − *u*, and *G*_2_′ = *G*_1_ − *v*.

By applying the definition of vertex-weighted Wiener number, we have

(5) WvG2∗−WvG1∗=dG1∗u+dG1∗v2+∑x∈G1′1+dG1x21+dG1x,u+∑y∈G2′1+dG2y21+dG2y,v+∑x∈G1′dG1x+dG1∗u+dG1∗v−12dG1x,u+∑y∈G2′dG2y+dG1∗u+dG1∗v−12dG2y,v+∑x∈G1′ ∑y∈G2′dG1x+dG2y2dG1∗x,y−1−dG1∗u+dG1∗v2−∑x∈G1′dG1x+dG1∗u2dG1x,u−∑y∈G2′dG2y+dG1∗v2dG2y,v−∑x∈G1′dG1x+dG1∗v2dG1x,u+1−∑y∈G2′dG2y+dG1∗u2dG2y,v+1−∑x∈G1′ ∑y∈G2′dG1x+dG2y2dG1∗x,y=1−dG1∗v2∑x∈G1′+1−dG1∗u2+∑y∈G2′−∑x∈G1′ ∑y∈G2′dG1x+dG2y2.

Hence, we get *W*_*v*_(*G*_2_^*∗*^) < *W*_*v*_(*G*_1_^*∗*^) in terms of *d*_*G*_1_^*∗*^_(*u*) ≥ 3 and *d*_*G*_1_^*∗*^_(*v*) ≥ 3.

We emphasize here that any bicyclic molecular graph can be changed into a bicyclic molecular graph with all the edges that are not on the cycles but pendant edges by repeating Transformation A. Moreover, the vertex-weighted Wiener number of the molecular graphs decreased after such changing.

**Figure 3:**
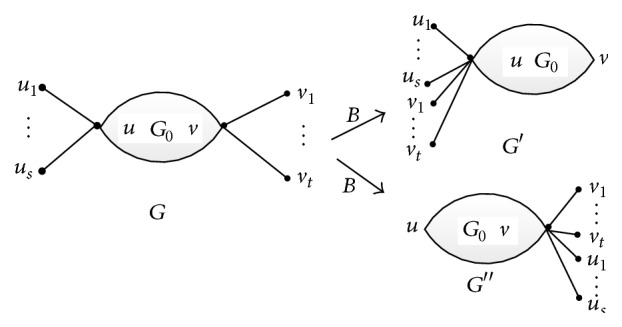
Transformation B.

*Transformation B*. Let *u* and *v* be two vertices in molecular graph *G*. Vertices *u*_1_, *u*_2_,…, *u*_*s*_ are the leaves adjacent to *u*, and vertices *v*_1_, *v*_2_,…, *v*_*t*_ are the leaves adjacent to *v*. Set *G*′ = *G* − {*vv*_1_, *vv*_2_,…, *vv*_*t*_}+{*uv*_1_, *uv*_2_,…, *uv*_*t*_}, *G*′′ = *G* − {*uu*_1_, *uu*_2_,…, *uu*_*s*_}+{*vu*_1_, *vu*_2_,…, *vu*_*s*_}, and |*V*(*G*_0_)|≥3. The explanation for Transformation B was presented in Figure 3.

**Lemma 2 .**

Let *G*′ and *G*′′ be molecular graphs deduced from *G* in view of Transformation B. Then, one infers *W*_*v*_(*G*) > *W*_*v*_(*G*′) or *W*_*v*_(*G*) > *W*_*v*_(*G*′′).

**Proof**

Let *G*_0_^*∗*^ = *G*_0_ − {*u*, *v*}. By means of the definition of vertex-weighted Wiener number, we yield

(6) WvG′−WvG=1+12×2s+t2 +∑x∈G0∗dG0∗x+dGu+t2dGx,u +∑x∈G0∗dG0∗x+dGv−t2dGx,v +dGu+dGv2dGu,v+s+t1+dGu+t2 +s+t1+dGv−t2dGu,v+1 +t∑x∈G0∗1+dG0∗x2dGx,u+1−2s2 −2t2−stdGu,v+2 −∑x∈G0∗dG0∗x+dGu2dGx,u −∑x∈G0∗dG0∗x+dGv2dGx,v−dGu+dGv2 ·dGu,v−s1+dGu2−t1+dGv2−s ·1+dGv2dGu,v+1−t1+dGu2dGu,v +dGu,v1−t∑x∈G0∗1+dG0∗x2dGx,v+1=s2+2st +t2−s−t+∑x∈G0∗dG0∗x2dGx,u+dGu2 ·∑x∈G0∗dGx,u+t2∑x∈G0∗dGx,u +∑x∈G0∗dG0∗x2dGx,v+dGv2∑x∈G0∗dGx,v−t2 ·∑x∈G0∗dGx,v+dGu+dGv2dGu,v +sdGu2+tdGu2+sdGu,v2+tsdGu,v2 +sdGv2+tdGv2+s2dGvdGu,v +t2dGvdGu,v−st2dGu,v−t22dGu,v +t∑x∈G0∗1+dG0∗x2dGx,u+1−s2−t2+s+t −2st−stdGu,v−∑x∈G0∗dG0∗x2dGx,u−dGu2 ·∑x∈G0∗dGx,u−∑x∈G0∗dG0∗x2dGx,v−dGv2 ·∑x∈G0∗dGx,v−dGu+dGv2dGu,v−s+t −sdGu2−tdGv2−sdGv2dGu,v −sdGu,v2−sdGv2−tdu2dGu,v−t2dGu, v−t2dGu−t∑x∈G0∗1+dG0∗x2dGx,v+1 =t∑x∈G0∗2+dG0∗x2dGx,u−dGx,v +∑x∈G0∗2+dG0∗x2dGx,u−dGx,vtdGu,vdGv−dGu2−t3s+t2dGu,v.

Similarly, we have

(7) WvG′′−WvG=s∑x∈G0∗2+dG0∗x2dGx,v−dGx,u+sdGu,vdGu−dGv2−s3t+s2dGu,v.

If *W*_*v*_(*G*′) − *W*_*v*_(*G*) ≥ 0, then we yield

(8) ∑x∈G0∗2+dG0∗x2dGx,u+dGv2dGu,v≥∑x∈G0∗2+dG0∗x2dGx,v+dGu2dGu,v+3s+t2dGu,v.

Thus,

(9) WvG′′−WvG=s∑x∈G0∗2+dG0∗x2dGx,v−dGx,u+dGu,vdGu−dGv2−3t+s2dGu,v≤−2ss+tdGu,v<0.

Otherwise, *W*_*v*_(*G*′′) − *W*_*v*_(*G*) ≥ 0; thus,

(10) ∑x∈G0∗2+dG0∗x2dGx,v+dGu2dGu,v≥∑x∈G0∗2+dG0∗x2dGx,u+dGv2dGu,v+3t+s2dGu,v.

Then,

(11) WvG′−WvG=t∑x∈G0∗2+dG0∗x2dGx,u−dGx,v+dGu,vdGv−dGu2−3s+t2dGu,v≤−2ts+tdGu,v<0.

Therefore, we complete the proof.

It is obvious that after repeating Transformation A, if continue repeating Transformation B, any bicyclic molecular graph can be changed into a new bicyclic molecular graph so that all the pendant edges are attached to the same vertex. Meanwhile, the vertex-weighted Wiener number of the graphs decreased after changing.

**Lemma 3 .**

Let *G*′ and *G*′′ be the molecular graphs described in Figure 3. Set *G*_0_^*∗*^ = *G*_0_ − {*u*, *v*}. Then, *W*_*v*_(*G*′) < *W*_*v*_(*G*′′) if *d*_*G*_0__(*u*) > *d*_*G*_0__(*v*) and ∑_*x*∈*G*_0_^*∗*^_ *d*_*G*_0_^*∗*^_(*x*)*d*_*G*_0_^*∗*^_(*x*, *u*) < ∑_*x*∈*G*_0_^*∗*^_ *d*_*G*_0_^*∗*^_(*x*)*d*_*G*_0_^*∗*^_(*x*, *v*); otherwise, *W*_*v*_(*G*′) > *W*_*v*_(*G*′′).

**Proof**

Using the definition of vertex-weighted Wiener number, we obtain

(12) WvG′=2s+t2+∑x∈G0∗dG0∗x+dG0v2dG0∗x,v+∑x∈G0∗dG0∗x+dG0u+s+t2dG0∗x,u+s+t·1+dG0u+s+t2+s+t·1+dG0v21+dG0u,v+dG0u+dG0v+s+t2dG0u,v+s+t·∑x∈G0∗1+dG0∗x21+dG0∗x,u·∑x,y∈G0∗dG0∗y+dG0∗x2dG0∗x,y,WvG′′=2s+t2+∑x∈G0∗dG0∗x+dG0u2dG0∗x,u+∑x∈G0∗dG0∗x+dG0v+s+t2dG0∗x,u+s+t·1+dG0v+s+t2+s+t·1+dG0u21+dG0u,v+dG0u+dG0v+s+t2dG0u,v+s+t·∑x∈G0∗1+dG0∗x21+dG0∗x,v+∑x,y∈G0∗dG0∗y+dG0∗x2dG0∗x,y.

Thus, we have

(13) WvG′−WvG′′=s+t·∑x∈G0∗2+dG0∗x2dG0∗x,u−dG0∗x,v+dG0u,vdG0v−dG0u2.

Therefore, one gets the desired result.

**Lemma 4 .**

**Figure 4:**
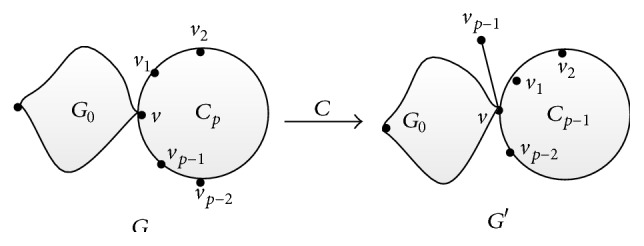
Transformation C.

Suppose that *G* is a molecular graph of order *n* ≥ 7 gotten from a connected molecular graph *G*_0_≇*P*_1_ and a cycle *C*_*p*_ = *v*_0_ *v*_1_ ⋯ *v*_*p*−1_ *v*_0_ (*p* ≥ 4 for *p* is even; otherwise *p* ≥ 5) by identifying *v*_0_ with a vertex *v* of the molecular graph *G*_0_ (see Figure 4 for more details). Let *G*′ = *G* − *v*_*p*−1_ *v*_*p*−2_ + *vv*_*p*−2_. This molecular graph operation is labelled as grafting Transformation C. Then, one deduces *W*_*v*_(*G*) > *W*_*v*_(*G*′).

**Proof**

For convenience, we set *G*_0_′ = *G*_0_ − *v*, *C*_*p*_′ = *C*_*p*_ − {*v*, *v*_*q*−1_}, and *C*_*p*−1_′ = *C*_*p*−1_ − *v*. We divided the proof into two cases according to the parity of *p*.

*Case 1* (*p* ≡ 0 (mod ⁡2)). Consider the following:

(14) WvG∑x,y∈G0′dG0′x+dG0′y2dG0′x,y+∑x∈G0′dG0′x+dGv2dG0′x,v+2∑x,y∈Cp′dCp′x,y+2+dGv2∑x∈Cp′dCp′x,v+2∑x∈Cp′dCp′x,v+1+dGv2+∑x∈G0′2+dG0′x2dG0′x,v+∑x∈G0′2+dG0′x2dG0′x,v+1=∑x,y∈G0′dG0′x+dG0′y2dG0′x,y+p∑x∈G0′dG0′x2dG0′x,v+dGv+2p−22∑x∈G0′dG0′x,v+p28∑x∈G0′dG0′x+p28dGv+p34−p24+p24VG0′−1.

Similarly, we have

(15) WvG=∑x,y∈G0′dG0′x+dG0′y2dG0′x,y+p∑x∈G0′dG0′x2dG0′x,v+dGv+2p−22∑x∈G0′dG0′x,v+p28−p4+12∑x∈G0′dG0′x+p28−p4+12dGv+p34−p22+3p2−2+p24−p2+12VG0′−1.

This implies

(16) WvG′−WvG12−p4∑x∈G0′dG0′x+12−p4dGv+12−p2VG0′−1−p−324+14<0.

The last step follows the form *p* ≥ 4.

*Case 2* (*p* ≡ 1 (mod ⁡2)). Similar to Case 1, we deduce

(17) WvG∑x,y∈G0′dG0′x+dG0′y2dG0′x,y+∑x∈G0′dG0′x+dGv2dG0′x,v+2∑x,y∈Cp′dCp′x,y+2+dGv2∑x∈Cp′dCp′x,v+2∑x∈Cp′dCp′x,v+1+dGv2+∑x∈G0′2+dG0′x2dG0′x,v+∑x∈G0′2+dG0′x2dG0′x,v+1=∑x,y∈G0′dG0′x+dG0′y2dG0′x,y+p2∑x∈G0′dG0′xdG0′x,v+dGv+2p−22∑x∈G0′dG0′x,v+p28−18∑x∈G0′dG0′x+p28−18dGv+p34−p24−p4+14+p24−14VG0′−1.

Similarly, we get

(18) WvG=∑x,y∈G0′dG0′x+dG0′y2dG0′x,y+p2∑x∈G0′dG0′xdG0′x,v+dGv+2p−22∑x∈G0′dG0′x,v+p28−p4+58∑x∈G0′dG0′x+p28−p4+58dGv+p34−p22+7p4−2+p24−p2+34VG0′−1.

Therefore,

(19) WvG′−WvG34−p4∑x∈G0′dG0′x+34−p4dGv+2−p2VG0′−1−p−424+74<0.

The last step is determined by *p* ≥ 5 when *p* is odd.

Thus, we complete the proof.

In the last part of this section, we present the previously known result as follows, which will be used in our proofs.

**Lemma 5 .**

Let *C*_*n*_ be the cycle of order *n*, and *v* is a vertex on *C*_*n*_. Then

(20) ∑x∈VCndCnv,x=14n2,if  n  is  even14n2−1,if  n  is  odd,WvCn=14n3,if  n  is  even14n3−n,if  n  is  odd.

In fact, the first equation in Lemma 5 is given by Buckley and Harary [21], and the second one can be obtained directly from the former equation.

## 3. Main Results and Proofs

In this section, we present lower bounds for the vertex-weighted Wiener number in bicyclic molecular graphs.

### 3.1. The Extremal Vertex-Weighted Wiener Number in Θ(*p*, *q*)

**Figure 5:**
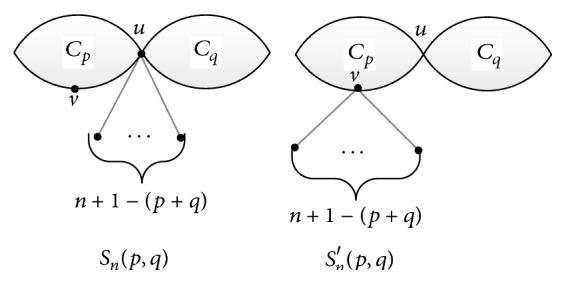
The molecular graphs *S*_*n*_(*p*, *q*) and *S*_*n*_′(*p*, *q*).

In this subsection, we determine the bicyclic molecular structure with the smallest vertex-weighted Wiener number in Θ(*p*, *q*). Let *S*_*n*_(*p*, *q*) be a molecular graph in Θ(*p*, *q*) so that *n* + 1 − (*p* + *q*) pendent edges are attached to the common vertex of *C*_*p*_ and *C*_*q*_, and let *S*_*n*_′(*p*, *q*) be a molecular graph in Θ(*p*, *q*) which satisfies that *n* + 1 − (*p* + *q*) pendent edges are attached to the vertex *v* of *C*_*p*_ or *C*_*q*_. See Figure 5 for the structure of these two molecular graphs.

**Theorem 6 .**

If *G* ∈ Θ(*p*, *q*) is a molecular graph of order *n*, then *W*_*v*_(*S*_*n*_(*p*, *q*)) < *W*_*v*_(*G*).

**Proof**

A new molecular graph *G*′ such that all the edges not on the cycles are the pendant edges attached to the same vertex *v* can be obtained in terms of repeating Transformations A and B on molecular graph *G*. In view of Lemmas 1 and 2, we have *W*_*v*_(*G*) ≥ *W*_*v*_(*G*′) with the equality if and only if all the edges that are not on the cycles are also the pendant edges attached to the same vertex in *G*. Let *u* be the common vertex of *C*_*p*_ and *C*_*q*_. Clearly, *v* ≠ *u* if *G*′≇*S*_*n*_(*p*, *q*).

Without loss of generality, we assume that *v* is on the cycle *C*_*p*_ and *G*′≅*S*_*n*_′(*p*, *q*) is manifested in Figure 5. Set *G*_0_ = *C*_*p*_ *uC*_*q*_ and *G*_0_^*∗*^ = *G*_0_ − {*u*, *v*} in *S*_*n*_(*p*, *q*) and *S*_*n*_′(*p*, *q*). We infer that *d*_*G*_0__(*u*) = 4 > *d*_*G*_0__(*v*) = 2 and ∑_*x*∈*G*_0_^*∗*^_ *d*_*G*_0_^*∗*^_(*x*)*d*_*G*_0_^*∗*^_(*x*, *u*) = 2∑_*x*∈*G*_0_^*∗*^_ *d*_*G*_0_^*∗*^_(*x*, *u*) < 2∑_*x*∈*G*_0_^*∗*^_ *d*_*G*_0_^*∗*^_(*x*, *v*).

By the proof of Lemma 3, we get *W*_*v*_(*S*_*n*_(*p*, *q*)) < *W*_*v*_(*S*_*n*_′(*p*, *q*)), and thus *W*_*v*_(*S*_*n*_(*p*, *q*)) < *W*_*v*_(*G*). This completes the proof.

**Theorem 7 .**

The following facts hold:

(1) *W*_*v*_(*S*_*n*_(*p*, *q*)) ≥ *W*_*v*_(*S*_*n*_(*p* − 1, *q*));
(2) for all *p* ≥ 3 and *q* ≥ 3, *S*_*n*_(3,3) is the unique graph with the smallest vertex-weighted Wiener number in Θ(*p*, *q*).

**Proof**

**Figure 6:**
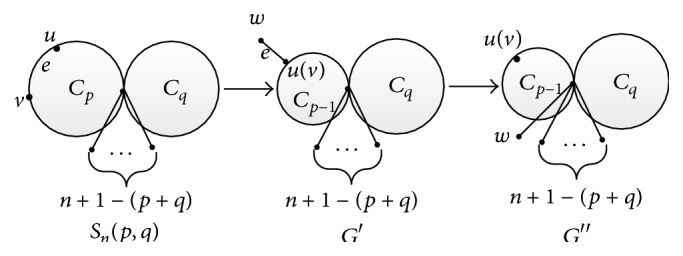
Graph transformations.

For the fixed *p* ≥ 3 and *q* ≥ 3, *S*_*n*_(*p*, *q*) is the unique molecular graph with the smallest vertex-weighted Wiener number in Θ(*p*, *q*) by the conclusion presented in Theorem 6. Let *e* = *uv* be an edge of cycle *C*_*p*_ (by the symmetry of *p* and *q*), using grafting Transformation A on *S*_*n*_(*p*, *q*). Then *S*_*n*_(*p*, *q*) can be changed into the molecular graph *G*′ and *e* is changed into a pendent edge adjacent to *u*(*v*) (see Figure 6 for the details of transformations). Thus, we obtain *W*_*v*_(*S*_*n*_(*p*, *q*)) > *W*_*v*_(*G*′) by applying Lemma 5. The molecular graph *G*′ can be changed into *G*′′ (*G*′′≅*S*_*n*_(*p* − 1, *q*)) by continuously applying Transformation C on *G*′, and we further get *W*_*v*_(*G*′) > *W*_*v*_(*G*′′).

In terms of repeating Transformations A, B, and C on *S*_*n*_(*p*, *q*), it can be changed into *S*_*n*_(3,3), and we further infer *W*_*v*_(*S*_*n*_(*p*, *q*)) > *W*_*v*_(*S*_*n*_(3,3)) if *p* ≥ 3 and *q* > 3 or *p* > 3 and *q* ≥ 3. Finally, we deduce *W*_*v*_(*S*_*n*_(*p*, *q*)) ≥ *W*_*v*_(*S*_*n*_(3,3)) for all molecular graphs in Θ(*p*, *q*) with *p* ≥ 3 and *q* ≥ 3, and the equality holds if and only if *G*≅*S*_*n*_(3,3).

In addition, by means of the definition of vertex-weighted Wiener number, we have

(21) WvSn3,32n−52+1+n−12n−5+1+22×2×n−5×4+2+22×2+2+n−12×4+2+22×2×4=3n2+n−162.

That is to say, the smallest vertex-weighted Wiener number in Θ(*p*, *q*) is (3*n* ^2^ + *n* − 16)/2, and the lower bound is obtained if and only if *G*≅*S*_*n*_(3,3).

### 3.2. The Extremal Vertex-Weighted Wiener Number in Γ(*p*, *q*)

**Figure 7:**
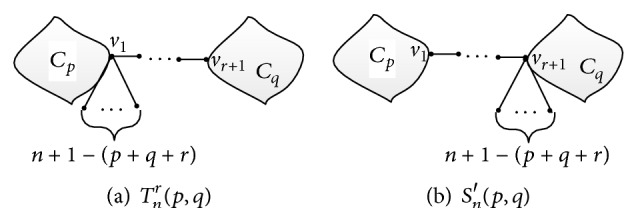
The molecular graphs *T*_*n*_^*r*^(*p*, *q*) and *T*_*n*_^*r*^(*q*, *p*).

In this part, we determine the bicyclic molecular graph with the smallest vertex-weighted Wiener number in Γ(*p*, *q*). Let *T*_*n*_^*r*^(*p*, *q*) be the molecular (*n*, *n* + 1)-graph generating by connecting *C*_*p*_ and *C*_*q*_ by a path of length *r* and the other *n* + 1 − *p* − *q* − *r* edges are all attached to the common vertex of the path and *C*_*p*_; see Figure 7(a).

Analogous to the discussion in former section, we have the following conclusions. We omit the proof here since the techniques to get the detail proofs are similar to the tricks presented in the former section.

**Theorem 8 .**

If *G* ∈ Γ(*p*, *q*), the length of the shortest path connecting *C*_*p*_ and *C*_*q*_ in *G* is *r*, then

(1) *W*_*v*_(*G*) ≥ *W*_*v*_(*T*_*n*_^*r*^(*p*, *q*)) with the equality if and only if *G*≅*T*_*n*_^*r*^(*p*, *q*); or
(2) *W*_*v*_(*G*) ≥ *W*_*v*_(*T*_*n*_^*r*^(*q*, *p*)) with the equality if and only if *G*≅*T*_*n*_^*r*^(*q*, *p*).

**Theorem 9 .**

If *r* ≥ 2, then *W*_*v*_(*T*_*n*_^*r*^(*p*, *q*)) < *W*_*v*_(*T*_*n*_^*r*−1^(*p*, *q*)).

**Theorem 10 .**

Consider the following: *W*_*v*_(*T*_*n*_^1^(*p*, *q*)) ≥ *W*_*v*_(*T*_*n*_^1^(3,3)) with the equality if and only if *p* = *q* = 3.

**Theorem 11 .**

The *W*_*v*_(*T*_*n*_^1^(3,3)) is the unique molecular graph with the smallest vertex-weighted Wiener number among all molecular graphs in Γ(*p*, *q*) for all *q* ≥ 3 and *p* ≥ 3.

Note that the vertex-weighted Wiener number of *W*_*v*_(*T*_*n*_^1^(3,3)) is (3*n* ^2^ + 11*n* − 52)/2, which is the smallest vertex-weighted Wiener number in Γ(*p*, *q*).

### 3.3. The Extremal Vertex-Weighted Wiener Number in *Ω*(*p*, *q*, *l*)

**Figure 8:**
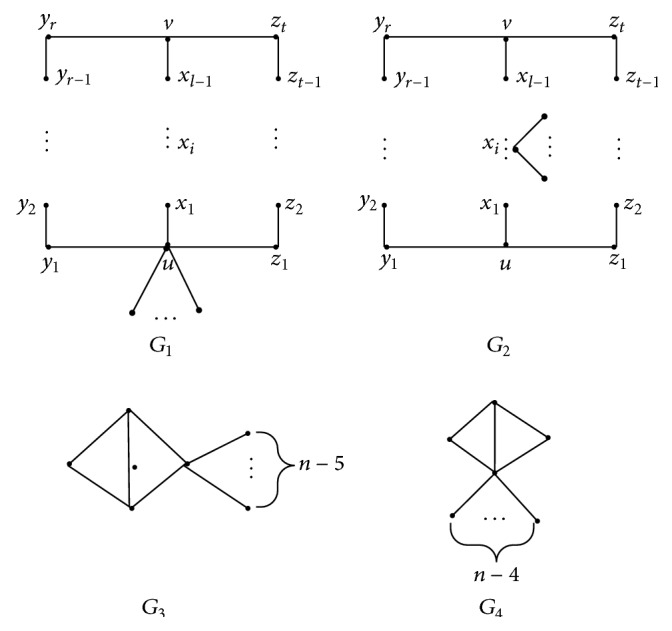
The molecular graphs *G*_*i*_ (*i* = 1,2, 3,4).

In this part, we derive the bicyclic molecular graph with the smallest vertex-weighted Wiener number in *Ω*(*p*, *q*, *l*). Let *θ*_*n*_^*l*^(*p*, *q*) be the molecular graph obtained from the molecular graph in Figure 1(c) by attaching *n* + 1 + *l* − (*p* + *q*) vertices to one of its vertices with degree 3 (see Figure 8 for more details).

**Theorem 12 .**

Let *G* ∈ *Ω*(*p*, *q*, *l*). Then *W*_*v*_(*G*) ≥ *W*_*v*_(*G*_4_) with the equality if and only if *G*≅*G*_4_, where *G*_4_ is the molecular graph presented in Figure 8.

**Proof**

A new molecular graph *G*′ with all the edges that are not on the cycles but are the pendant edges being attached to the same vertex *v*_0_ can be obtained via repeating the Transformations A and B on molecular graph *G*; that is, *G*′ is one of the molecular graphs in Figure 8. By means of Lemmas 2 and 3, we get *W*_*v*_(*G*) ≥ *W*_*v*_(*G*′) with the equality if and only if all the edges that are not on the cycles are also the pendant edges attached to the same vertex in *G*.

Let *W*_1_ = *ux*_1_ *x*_2_ ⋯ *x*_*l*−1_ *v* be the common path of *C*_*p*_ and *C*_*q*_ of the molecular graph *G*′, *W*_2_ = *uy*_1_ *y*_2_ ⋯ *y*_*r*_ *v* and *W*_3_ = *uz*_1_ *z*_2_ ⋯ *z*_*t*_ *v* be the other paths are from *u* to *v* on *C*_*p*_ and *C*_*q*_, respectively. Here, *r* = *p* − *l* − 1, *t* = *q* − *l* − 1, *r* ≥ 0, *t* ≥ 0, *l* ≥ 1, and *r* + *t* + *l* ≥ 3.

If there exists an edge *e* = *xy* in *G*_1_ satisfying that the degrees of *x* and *y* are both equal 2, then a molecular graph *G*_1_′ can be obtained in terms of contracting edge *e* and attaching a pendant edge *e*′ = *uu*′ to *u*, and we further have *W*_*v*_(*G*_1_) > *W*_*v*_(*G*_1_′) according to Lemma 2; repeating Transformation B on *G*_1_′, *G*_1_′ is changed into the molecular graph *G*_1_′′ such that the pendent edge is transferred to the vertex *u* and *W*_*v*_(*G*_1_′) > *W*_*v*_(*G*_1_′′) by Lemma 3. Continuously repeating these operations, the molecular graph *G* will finally be changed into *G*_4_. Consider that *W*_*v*_(*G*) ≥ *W*_*v*_(*G*_4_), and the equality holds if and only if *G*≅*G*_4_.

If there are two edges *e*_1_ and *e*_2_ in *G*_2_ so that the degrees of their end-vertices are equal to two, then we can obtain a molecular graph *G*_2_′ by means of contracting the edges *e*_1_ and *e*_2_ and attaching two pendant edges to *x*_*i*_; or if there exists an edge *e* in *G*_2_ such that the degrees of its end-vertices and *e*_2_ are equal to 2, a molecular graph *G*_2_′ can be obtained by contracting the edge *e* and attaching a pendant edge to *x*_*i*_. Using Lemmas 2 and 3, we get *W*_*v*_(*G*_2_) > *W*_*v*_(*G*_2_′). By continuously repeating the operations above, we verify that *W*_*v*_(*G*_2_) ≥ *W*_*v*_(*G*_3_) and equality holds if and only if *G*≅*G*_3_.

Furthermore, it is easy to calculate that *W*_*v*_(*G*_3_) = (3*n* ^2^ + 11*n* − 64)/2 and *W*_*v*_(*G*_4_) = (3*n* ^2^ + *n* − 18)/2. Thus, *W*_*v*_(*G*_3_) − *W*_*v*_(*G*_4_) = 5*n* − 23 > 0 since *n* ≥ 5.

Noting that *θ*_*n*_^1^(3,3)≅*G*_4_, we will use this notion in the last subsection.

### 3.4. Bicyclic Graphs with the Extremal Vertex-Weighted Wiener Number

Finally, we give the bicyclic molecular graph the smallest vertex-weighted Wiener number.

**Theorem 13 .**

*θ*_*n*_^1^(3,3) is the unique graph with the smallest vertex-weighted Wiener number among all bicyclic molecular graphs with *n*  (*n* ≥ 6) vertices.

**Proof**

**Figure 9:**
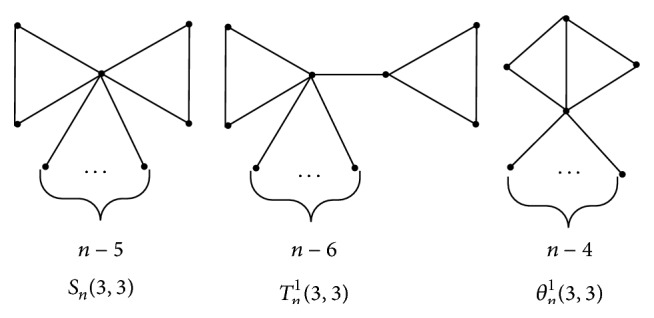
The molecular graphs *S*_*n*_(3,3), *T*_*n*_^1^(3,3), and *θ*_*n*_^1^(3,3).

By means of Theorems 7, 11, and 12, we only need to compare the vertex-weighted Wiener number of *S*_*n*_(3,3), *T*_*n*_^1^(3,3), and *θ*_*n*_^1^(3,3) (the structures of these three molecular graphs can be referred to in Figure 9).

By immediately computing, we have *W*_*v*_(*T*_*n*_^1^(3,3)) > *W*_*v*_(*S*_*n*_(3,3)) > *W*_*v*_(*θ*_*n*_^1^(3,3)) if *n* ≥ 6. Therefore, *θ*_*n*_^1^(3,3) has the smallest vertex-weighted Wiener number among all bicyclic molecular graphs with *n* vertices.

## 4. Conclusion

In our paper, we mainly report the extremal vertex-weighted Wiener number of bicyclic molecular graph with the help of molecular graph structural analysis and mathematical derivation. The vertex-weighted Wiener number is widely used in the analysis of both the melting point and boiling point for chemical compounds and QSPR/QSAR study. Thus, the promising prospects of the application for the chemical and pharmacy engineering will be illustrated in the theoretical conclusion that is obtained in this paper.
